# Warfarin for Left Ventricular Thrombus in Nonischemic Cardiomyopathy With Acute Kidney Injury: A Case Report and Literature Review of Anticoagulation Strategies in Renal Impairment

**DOI:** 10.7759/cureus.116513

**Published:** 2026-09-18

**Authors:** Sean Pack, Vikramjit Chhokar

**Affiliations:** 1 Internal Medicine, St. Francis - Emory Healthcare, Columbus, USA; 2 Cardiology, St. Francis - Emory Healthcare, Columbus, USA

**Keywords:** acute kidney injury, anticoagulation, cardiorenal syndrome, direct oral anticoagulants, heart failure, left ventricular thrombus, length of stay, nonischemic cardiomyopathy, warfarin

## Abstract

Left ventricular (LV) thrombus is a serious complication of cardiomyopathy with severely reduced ejection fraction, carrying significant risk of systemic embolization. While direct oral anticoagulants (DOACs) have emerged as a reasonable alternative to warfarin for LV thrombus treatment in the general population, no guideline-directed evidence exists for their use in patients with concomitant acute kidney injury (AKI) or significant renal impairment in this specific clinical context. We present the case of a 47-year-old male with newly diagnosed nonischemic cardiomyopathy (ejection fraction 25%), LV apical thrombus, and Kidney Disease: Improving Global Outcomes (KDIGO)-defined AKI attributed to type 1 cardiorenal syndrome, who was managed with heparin bridging to warfarin due to the absence of evidence supporting DOAC use for LV thrombus in the setting of renal dysfunction. The requirement for heparin-to-warfarin bridging prolonged the patient's hospitalization by five days, illustrating the tangible clinical and resource burden imposed by the current guideline gap. Chronic kidney disease (CKD) is present in approximately 45% of patients with heart failure with reduced ejection fraction; roughly two-thirds of heart failure hospitalizations are associated with a discharge estimated glomerular filtration rate below 60 mL/min/1.73 m², and AKI complicates approximately one-third of heart failure admissions, so this clinical scenario is far from rare. We additionally provide a literature review of the current evidence comparing DOACs and vitamin K antagonists (VKAs) for LV thrombus, DOAC pharmacokinetics in renal impairment, and the guideline landscape at this clinical intersection. Notably, every randomized trial comparing DOACs with warfarin for LV thrombus to date has explicitly excluded patients with moderate-to-severe renal impairment. This case and review underscore the need for prospective studies evaluating DOAC safety and efficacy for LV thrombus in patients with impaired renal function, which could reduce unnecessary hospital days and healthcare costs while maintaining safe anticoagulation.

## Introduction

Left ventricular (LV) thrombus remains a clinically significant complication in patients with severely reduced LV ejection fraction (LVEF), occurring in both ischemic and nonischemic cardiomyopathy. The risk of systemic thromboembolism, including ischemic stroke, necessitates prompt anticoagulation upon diagnosis. Vitamin K antagonists (VKAs), predominantly warfarin, have been the traditional standard of care for LV thrombus treatment, with at least three to six months of oral anticoagulation generally recommended [1].

In recent years, direct oral anticoagulants (DOACs) have gained traction as an alternative to warfarin for LV thrombus management. A meta-analysis conducted for the 2022 American Heart Association (AHA) Scientific Statement, pooling data from 21 studies and 3,057 patients, found no significant differences between DOACs and VKAs in stroke or systemic embolization (odds ratio (OR) 0.94; 95% confidence interval (CI), 0.70-1.25), all-cause mortality, thrombus resolution, or bleeding complications. That statement considers DOACs a reasonable alternative to VKAs for LV thrombus treatment. Importantly, DOACs offer a practical advantage: they can be initiated without the need for parenteral bridging anticoagulation, potentially allowing earlier hospital discharge [1].

However, a critical gap exists at the intersection of LV thrombus management and renal impairment. The AHA Scientific Statement explicitly notes that no data exist on the use of DOACs for either prophylaxis or treatment of LV thrombus in patients with end-stage renal disease, and that the choice of oral anticoagulant in such patients must rest on clinical judgment and shared decision-making [1]. While renal-stratified DOAC dosing recommendations exist for atrial fibrillation in the setting of chronic kidney disease (CKD) [2], these have not been validated for the LV thrombus indication, as no prospective study has evaluated renal-stratified DOAC dosing in this population. Furthermore, the pivotal DOAC trials for atrial fibrillation excluded patients with severe renal impairment; the ARISTOTLE trial, for example, excluded patients with a serum creatinine above 2.5 mg/dL or a calculated creatinine clearance below 25 mL/min [3]. A 2024 Journal of the American College of Cardiology (JACC) State-of-the-Art Review titled "When Direct Oral Anticoagulants Should Not Be Standard Treatment" explicitly categorizes LV thrombus among indications where DOAC safety and efficacy remain uncertain, noting that the three earliest randomized controlled trials (RCTs) were insufficiently powered, with a pooled sample of only 139 patients [4]. The situation is further complicated in patients with acute kidney injury (AKI), in whom fluctuating renal function renders DOAC pharmacokinetics unpredictable [4].

We present a case that illustrates this guideline gap and its downstream consequences: a patient with newly diagnosed nonischemic cardiomyopathy, LV apical thrombus, and concurrent AKI in whom warfarin was the only viable anticoagulant option, resulting in a prolonged hospitalization of five additional days solely for heparin-to-warfarin bridging and international normalized ratio (INR) optimization. We additionally provide a literature review of the evidence at this clinical intersection.

## Case presentation

A 47-year-old male with no significant prior medical history presented to the emergency department with progressive dyspnea on exertion, orthopnea, and bilateral lower extremity edema worsening over several weeks. He also endorsed early satiety, likely attributable to splanchnic congestion from volume overload. He denied chest pain, palpitations, or acute dyspnea at rest while sitting upright and did not appear to be in acute distress.

A directed history was obtained to evaluate potentially reversible etiologies of cardiomyopathy. He reported drinking two to three alcoholic beverages per week socially, an intake well below the threshold associated with alcohol-induced cardiomyopathy. He had smoked approximately one pack of cigarettes per week and had quit two weeks prior to presentation. He denied any recreational or illicit drug use, including cocaine and other stimulants. He was taking no prescription or over-the-counter medications, including nonsteroidal anti-inflammatory drugs. He reported no prior chemotherapy or chest radiation, no family history of cardiomyopathy, heart failure, or sudden cardiac death, and no preceding viral illness. He had never established care with a physician and had no prior laboratory testing, which accounts for the absence of any documented baseline renal function.

Initial vital signs were notable for a blood pressure of 189/132 mmHg, heart rate of 88 beats per minute, and oxygen saturation of 99% on room air. Physical examination revealed 3+ pitting edema to the knees bilaterally and bibasilar crackles on pulmonary auscultation.

Initial laboratory evaluation was notable for a white blood cell count of 15.6 × 10³/µL with neutrophilic predominance, B-type natriuretic peptide (BNP) of 3,292 pg/mL, initial high-sensitivity troponin I of 773.9 ng/L, serum creatinine of 2.6 mg/dL with unknown baseline, and serum albumin of 2.6 g/dL (Table 1).

**Table 1:** Admission laboratory evaluation demonstrating acute kidney injury, elevated cardiac biomarkers, and laboratory findings consistent with acute decompensated heart failure. ALT, alanine aminotransferase; AST, aspartate aminotransferase; BNP, B-type natriuretic peptide; BUN, blood urea nitrogen; H, high; L, low; MCH, mean corpuscular hemoglobin; MCHC, mean corpuscular hemoglobin concentration; MCV, mean corpuscular volume; MDW, monocyte distribution width; MPV, mean platelet volume; RBC, red blood cell; RDW, red cell distribution width; WBC, white blood cell

Laboratory Test	Result	Reference Range
Complete Blood Count		
WBC (×10³/µL)	15.60 (H)	4.0-11.0
RBC (×10⁶/µL)	5.09	4.2-5.9
Hemoglobin (g/dL)	14.6	12.0-17.5
Hematocrit (%)	44.2	36-53
MCV (fL)	87	80-100
MCH (pg)	28.6	27-33
MCHC (g/dL)	33	32-36
RDW (%)	14.4	11.5-14.5
Platelets (×10³/µL)	268	150-400
MPV (fL)	8.6	7.5-11.5
Neutrophils (%)	78.5 (H)	40-70
Lymphocytes (%)	9.9 (L)	20-40
Monocytes (%)	10.8 (H)	2-8
Eosinophils (%)	0.1 (L)	1-4
Basophils (%)	0.7	0-1
MDW	19.9	<20.0
Routine Chemistry		
Sodium (mmol/L)	132 (L)	135-145
Potassium (mmol/L)	4	3.5-5.0
Chloride (mmol/L)	102	98-107
CO₂ (mmol/L)	18 (L)	22-29
Alkaline Phosphatase (U/L)	139 (H)	35-120
AST (U/L)	133 (H)	10-40
ALT (U/L)	150 (H)	7-56
BUN (mg/dL)	43 (H)	7-20
Glucose (mg/dL)	104	70-99
Creatinine (mg/dL)	2.6 (H)	0.6-1.3
eGFR (mL/min/1.73 m²)	29 (L)	≥60
Calcium (mg/dL)	8.0 (L)	8.6-10.2
Total Protein (g/dL)	4.7 (L)	6.4-8.3
Albumin (g/dL)	2.6 (L)	3.5-5.0
Total Bilirubin (mg/dL)	2.3 (H)	0.2-1.2
Anion Gap (mmol/L)	12	8-16
Cardiac Markers		
BNP (pg/mL)	3,292 (H)	<100
hs-Troponin I, Baseline (ng/L)	773.9 (H)	<34*
hs-Troponin I, 1 Hour (ng/L)	648.5 (H)	<34*
hs-Troponin I, 3 Hours (ng/L)	714 (H)	<34*

Urinalysis demonstrated light-yellow, clear urine with a pH of 6.5 and specific gravity of 1.012, negative for blood, protein, nitrite, leukocyte esterase, ketones, and bilirubin, with normal glucose and a urobilinogen of 2 mg/dL consistent with the concurrent hyperbilirubinemia and hepatic congestion. Despite the negative dipstick protein, quantitative testing revealed a urine protein of 16 mg/dL against a urine creatinine of 23.9 mg/dL, yielding a urine protein-to-creatinine ratio of approximately 670 mg/g; the discordance reflects the well-recognized insensitivity of dipstick protein in dilute urine. This degree of proteinuria is consistent with either congestion-associated proteinuria, which is described in acute decompensated heart failure and frequently attenuates with decongestion, or an underlying CKD unmasked by the acute illness.

Retroperitoneal ultrasonography demonstrated mild heterogeneity of both kidneys without hydronephrosis or focal abnormality, together with a small amount of intraluminal debris within the urinary bladder. The absence of hydronephrosis excluded an obstructive etiology, while the parenchymal heterogeneity, taken with the quantitative proteinuria, raised the possibility of an unrecognized chronic component underlying the acute injury.

Serum protein electrophoresis with immunofixation demonstrated no monoclonal protein, with alpha-1 globulin 0.3 g/dL, alpha-2 globulin 0.7 g/dL, beta globulin 0.7 g/dL, gamma globulin 0.6 g/dL, and total globulin 2.4 g/dL; the uniformly reduced fractions were consistent with the patient's global hypoproteinemia. Serum free light chains were not performed. Thyroid function testing, iron studies, HIV serology, and antinuclear antibody testing were not obtained, so the etiologic evaluation rested on the clinical history, coronary angiography, and exclusion of a monoclonal process.

Electrocardiogram (ECG) demonstrated normal sinus rhythm, possible left atrial enlargement, and septal infarct of undetermined age (Figure 1).

**Figure 1:**
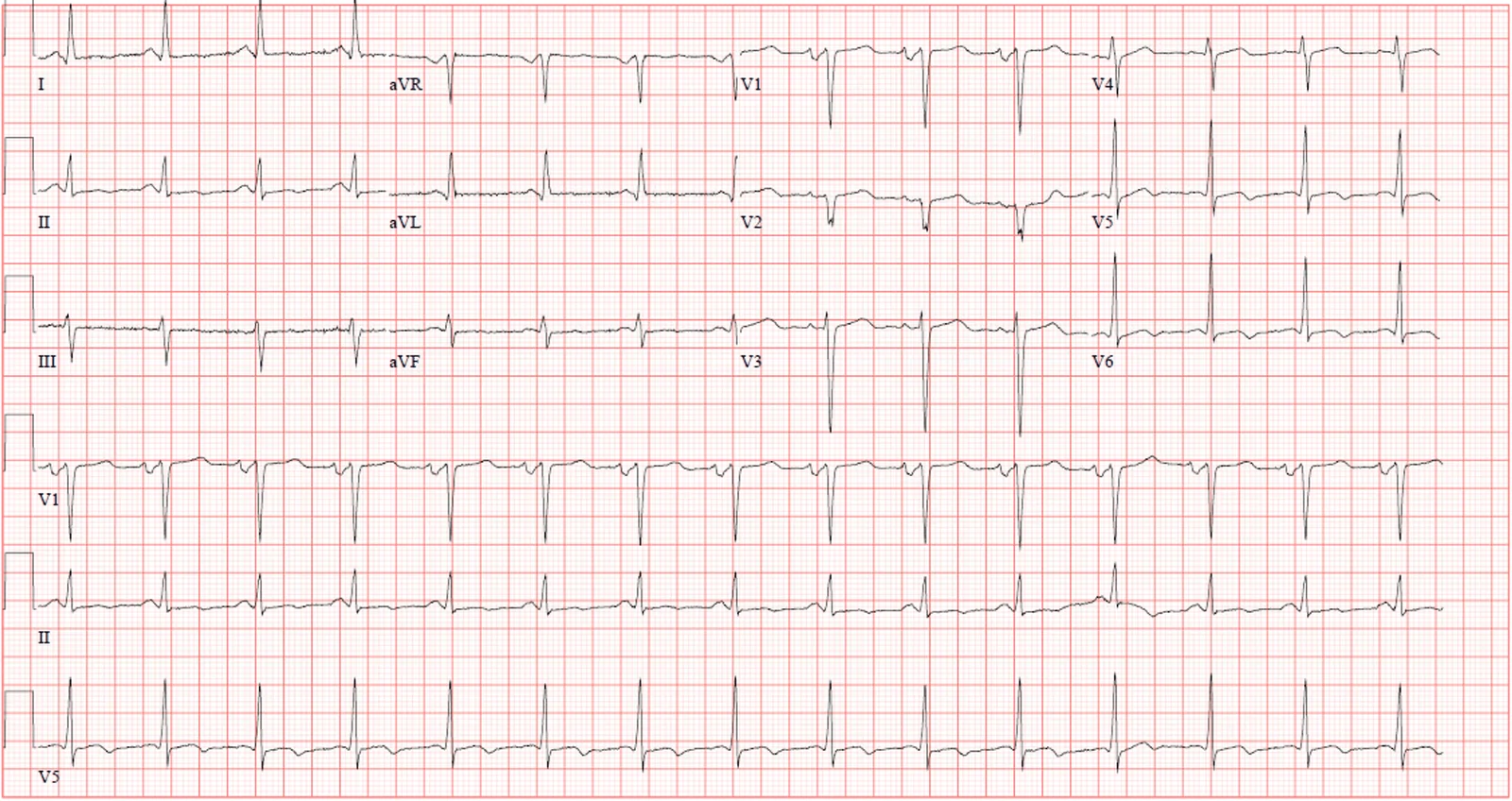
Admission electrocardiogram demonstrating normal sinus rhythm with possible left atrial enlargement and septal infarct of undetermined age.

Transthoracic echocardiography revealed a dilated LV cavity with increased wall thickness and severely reduced systolic function with an estimated ejection fraction of 25%. Doppler parameters were consistent with a reversible restrictive filling pattern, indicative of decreased LV diastolic compliance and/or increased left atrial pressure (grade 3 diastolic dysfunction). A thrombus was identified in the apex (Figure 2).

**Figure 2:**
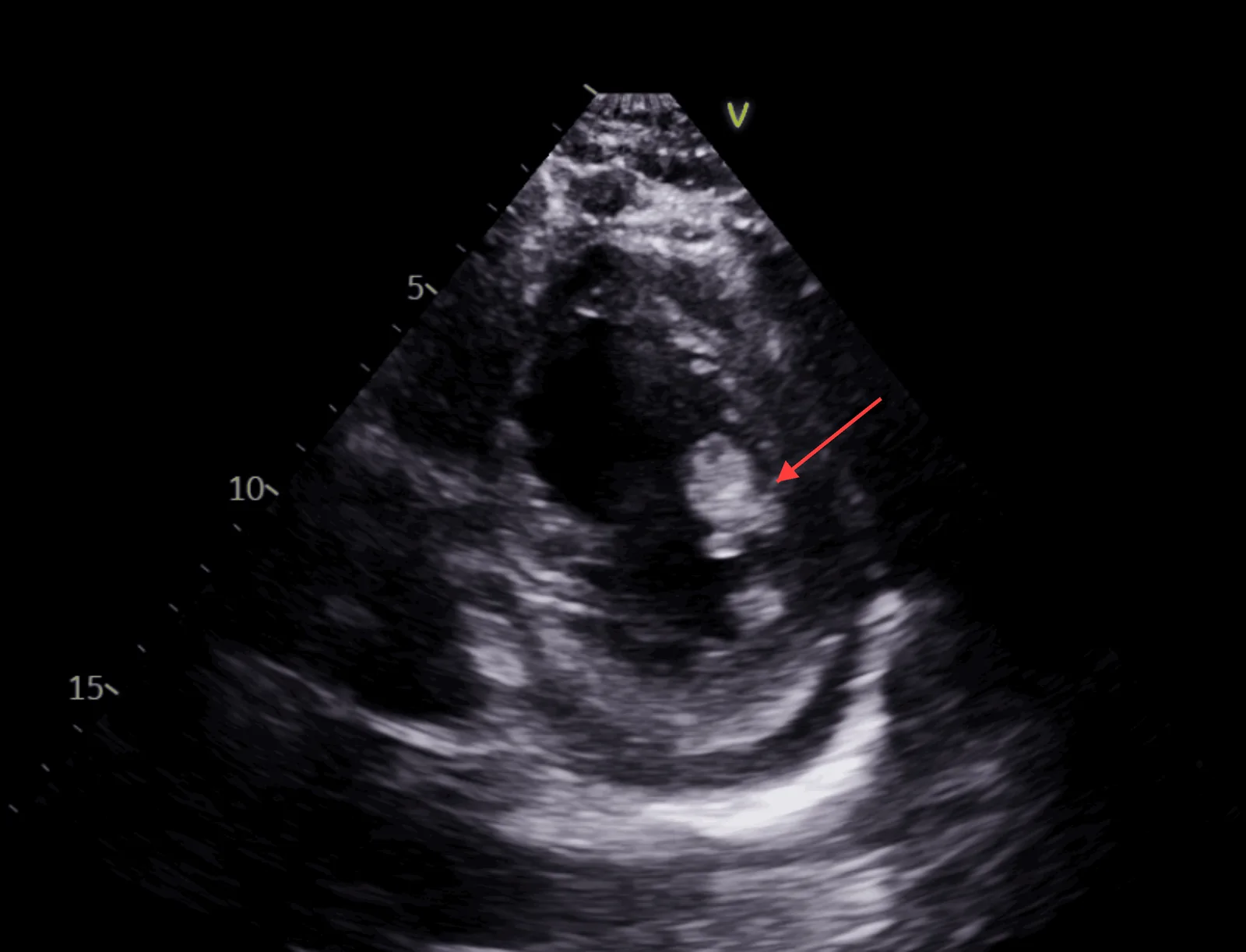
Transthoracic echocardiogram demonstrating a left ventricular apical thrombus in the setting of severe systolic dysfunction (left ventricular ejection fraction (LVEF) 25%).

The right ventricle was dilated with reduced systolic function and moderately increased systolic pressure. The left atrium was moderately to severely dilated, and the right atrium was moderately dilated. Valvular assessment demonstrated normal mitral valve leaflets with trivial regurgitation, a probably trileaflet aortic valve with no evidence of stenosis or regurgitation, and normal tricuspid valve leaflets with moderate regurgitation. No significant pulmonic regurgitation was noted. The aorta was not dilated. A trivial pericardial effusion was present, and the inferior vena cava was dilated, consistent with elevated right atrial pressure.

The patient was admitted for acute decompensated heart failure with a working differential of concomitant type 2 myocardial injury versus acute coronary syndrome. Intravenous furosemide was initiated at 40 mg every eight hours with strict intake and output monitoring, daily weights, and a goal of net negative 2-3 L per day. Sodium and fluid restriction were implemented. Acetazolamide, which has become an accepted adjunct for decongestion in acute decompensated heart failure, was considered but deferred given a serum bicarbonate of 18 mmol/L. Intravenous labetalol was administered for hypertensive emergency, and carvedilol was initiated and subsequently uptitrated to 25 mg twice daily given persistent hypertension. A heparin infusion was started for anticoagulation of the LV thrombus.

Left heart catheterization was performed on hospital day 2, when the serum creatinine was 2.5 mg/dL. The decision to proceed during the index admission, rather than deferring until renal recovery, rested on the presence of an LV apical thrombus in a patient with newly diagnosed LVEF 25% and no prior medical history: distinguishing ischemic from nonischemic etiology was necessary to determine anticoagulation duration, to establish whether revascularization or secondary prevention was indicated, and to guide initiation of guideline-directed medical therapy. Deferring the study would have required either discharging the patient on empiric anticoagulation without an etiologic diagnosis or extending the admission further, and the anticoagulation decision itself could not be finalized without it.

Peri-procedural measures to limit contrast-associated kidney injury included holding intravenous furosemide on the morning of the procedure, avoidance of other nephrotoxic agents, and deliberate minimization of contrast volume. A total of 80 mL of iso-osmolar iodinated contrast was administered, corresponding to a contrast volume-to-eGFR ratio of 2.8. Left ventriculography was not performed, both because of the known LV thrombus and to limit contrast exposure.

Coronary angiography demonstrated no significant coronary artery disease: the left main, left anterior descending, circumflex, and right coronary arteries were all without significant stenosis. LV end-diastolic pressure was elevated at 25 mmHg. These findings established a nonischemic etiology.

Renal function did not deteriorate following the procedure. Serum creatinine was 2.5 mg/dL on the day of catheterization, 2.0 mg/dL at 24 hours, 1.9 mg/dL at 48 hours, and 2.0 mg/dL at 72 hours, a trajectory that excludes contrast-associated AKI by Kidney Disease: Improving Global Outcomes (KDIGO) criteria and instead reflects continued response to decongestion (Table 2).

**Table 2:** Daily serum creatinine trend during hospitalization.

Hospital Day	Creatinine (mg/dL)	Notes
1	2.6	Admission
2	2.5	Left heart catheterization
3	2	24-hour post-catheterization
4	1.9	48-hour post-catheterization
5	2	72-hour post-catheterization
6	2	Nadir
7	1.8	-
8	1.8	-
9	1.8	Discharge

Serial troponin measurements showed a fluctuating pattern (773.9 → 648.5 → 714 ng/L) rather than a classic rise-and-fall, consistent with type 2 myocardial injury in the setting of volume overload and renal dysfunction rather than acute coronary syndrome. This interpretation was confirmed by the absence of obstructive coronary artery disease on catheterization. The leukocytosis improved without antibiotic therapy, consistent with reactive leukocytosis secondary to the acute heart failure exacerbation rather than an infectious etiology.

Serum creatinine on admission was 2.6 mg/dL. Although no baseline value was available, and an unknown baseline does not itself establish AKI, the diagnosis was supported by KDIGO criteria on the basis of the subsequent trajectory: creatinine fell by 0.9 mg/dL to a nadir of 1.7 mg/dL by hospital day 6 in direct temporal association with decongestion, a magnitude and rapidity of improvement incompatible with fixed CKD and indicating that the admission value substantially exceeded the patient's true baseline. The urinary sediment showed no hematuria, pyuria, or dipstick proteinuria, arguing against an active glomerular or acute interstitial process, and renal ultrasonography excluded obstruction. The clinical picture was therefore most consistent with type 1 cardiorenal syndrome, in which acute decompensated heart failure drives renal dysfunction through renal venous congestion, decreased renal perfusion, and neurohormonal activation [5]. The quantitative proteinuria of approximately 670 mg/g and the mild parenchymal heterogeneity on ultrasound nonetheless raise the possibility of a superimposed chronic component, which could not be resolved during the admission and required outpatient reassessment.

The daily creatinine trend is presented in Table 2. Renal function did not improve monotonically: creatinine declined from 2.6 to 1.9 mg/dL over the first four hospital days, rose to 2.0 mg/dL on day 5, fell to a nadir of 1.7 mg/dL on day 6, and then settled at 1.8 mg/dL for the final three hospital days. This day-to-day variability of 0.1 to 0.3 mg/dL during the first week, occurring in the setting of active diuresis and shifting volume status, is clinically important to the present case because it coincided precisely with the window in which an anticoagulant had to be selected.

With aggressive diuresis, the patient achieved a urine output of 4,425 mL over 24 hours. Nephrology was consulted and adjusted the diuretic regimen to furosemide 40 mg intravenously twice daily. The reduction in dosing frequency was undertaken because the patient had demonstrated a brisk natriuretic response with more than 4 L of urine output in 24 hours alongside concurrent improvement in creatinine; the aim was to consolidate decongestion at a more controlled rate while avoiding over-diuresis and a superimposed prerenal insult in a patient whose renal reserve was already compromised. Hypoalbuminemia (albumin 2.6 g/dL) was noted as a contributor to impaired diuretic delivery and was factored into the interpretation of diuretic responsiveness. Spironolactone was initiated by nephrology for mineralocorticoid receptor antagonism as a component of guideline-directed medical therapy in heart failure with reduced ejection fraction (HFrEF), with the additional near-term benefits of diuretic synergy and potassium conservation during high-dose loop diuresis; potassium and creatinine were monitored closely, and serum potassium remained within the normal range throughout. Sodium-glucose cotransporter-2 inhibitor and angiotensin receptor-neprilysin inhibitor therapy were deferred pending further renal recovery.

The identification of an LV apical thrombus on echocardiography necessitated therapeutic anticoagulation. The patient's concurrent AKI, with creatinine ranging from 1.7 to 2.6 mg/dL and corresponding estimated glomerular filtration rates (eGFRs) spanning CKD stage 3 to stage 4 territory during the hospitalization, posed a significant challenge for anticoagulant selection. While DOACs have been shown to be noninferior to warfarin for LV thrombus treatment in the general population, the 2022 AHA Scientific Statement explicitly acknowledges the absence of data on DOAC use for LV thrombus in patients with significant renal disease [1]. The 2024 JACC State-of-the-Art Review further categorizes LV thrombus among indications where DOAC safety and efficacy remain uncertain [4]. The pivotal DOAC trials for atrial fibrillation excluded patients with severe renal impairment [3], and DOAC dosing recommendations for CKD have not been validated in the LV thrombus population [1,2]. Critically, the day-to-day variability documented in Table 2 meant that any renal-based DOAC dose calculation would have been valid only for the day on which it was performed.

Given these considerations, the decision was made to continue the heparin infusion and bridge to warfarin with a target INR of 2.0-3.0. Warfarin was selected because it does not require renal dose adjustment, its anticoagulant effect can be reliably monitored via INR, it can be reversed if needed, and it remains the best-studied anticoagulant for LV thrombus in the setting of renal impairment.

Achieving a stable therapeutic INR proved challenging. The INR trend during hospitalization is presented in Table 3.

**Table 3:** Daily international normalized ratio (INR) trend during heparin-to-warfarin bridging demonstrating delayed achievement of therapeutic anticoagulation.

Hospital Day	INR
Day 2	1.42
Day 3	1.41
Day 4	1.29
Day 5	1.36
Day 6	1.28
Day 7	2
Day 8	1.47
Day 9	2.2

The INR remained subtherapeutic for the majority of the inpatient stay despite serial warfarin dose adjustments, only reaching the therapeutic range of 2.0-3.0 on the final hospital day. The heparin infusion was maintained as bridging anticoagulation throughout this period. This process prolonged the patient's hospitalization by five days beyond hospital day 4, at which point the patient was otherwise clinically stable for discharge: the heart failure had been adequately decongested, renal function had improved from 2.6 to 1.9 mg/dL and continued to improve thereafter, and the patient had no other acute medical needs requiring inpatient management.

The patient was discharged in stable condition on warfarin with close outpatient follow-up arranged at the cardiology INR clinic for ongoing INR monitoring and warfarin dose titration to a goal INR of 2.0-3.0. Nephrology follow-up was arranged at two weeks for reassessment of renal function once euvolemic, establishment of a true baseline creatinine, repeat quantification of proteinuria to determine whether the elevated protein-to-creatinine ratio reflected transient congestion or underlying CKD, and formal CKD staging if the creatinine failed to normalize. He was instructed to follow up with cardiology for continued management of his nonischemic cardiomyopathy and optimization of guideline-directed medical therapy as renal function allowed, including reconsideration of sodium-glucose cotransporter-2 inhibitor and angiotensin receptor-neprilysin inhibitor therapy, and for repeat echocardiography to assess thrombus resolution and recovery of systolic function. At the time of discharge, the patient had no active complaints.

## Discussion

This case highlights a significant and commonly encountered gap in current anticoagulation guidelines regarding DOAC use for LV thrombus in patients with renal dysfunction, and demonstrates the tangible downstream consequences of this gap.

A literature review was conducted using PubMed/MEDLINE, selected as the primary database given the scope of this narrative review, to identify evidence relevant to anticoagulation strategies for LV thrombus, with particular attention to the intersection with renal impairment and heart failure. The search covered articles published between 2011 and 2026 and was conducted in July 2026. Searches combined the terms "left ventricular thrombus," "direct oral anticoagulants," "vitamin K antagonists," "warfarin," "chronic kidney disease," "acute kidney injury," "heart failure with reduced ejection fraction," "renal pharmacokinetics," and "KDIGO." Reference lists of retrieved guidelines and reviews were also hand-searched to identify additional relevant articles. Inclusion criteria were: (1) scientific statements or clinical practice guidelines from major cardiovascular or nephrology societies; (2) meta-analyses or RCTs directly comparing DOACs and VKAs for LV thrombus; and (3) large observational studies or registry analyses addressing renal function and outcomes in heart failure populations. Case reports other than the index case and non-English-language articles were excluded. This was a narrative rather than systematic review; no formal risk-of-bias assessment was performed.

The evidence base comparing DOACs and VKAs for LV thrombus has expanded considerably in recent years, though it remains limited by small sample sizes and predominantly retrospective or observational study designs. The meta-analysis accompanying the 2022 AHA Scientific Statement pooled data from 21 studies encompassing 3,057 patients with LV thrombus (824 DOAC, 2,233 VKA) and found no significant differences between DOACs and VKAs in stroke or systemic embolization (OR 0.94; 95% CI, 0.70-1.25; P = 0.65), all-cause mortality (OR 0.92; 95% CI, 0.64-1.30; P = 0.63), thrombus resolution (OR 1.21; 95% CI, 0.89-1.64; P = 0.22), or bleeding complications (OR 0.79; 95% CI, 0.56-1.11; P = 0.17) [1]. A pooled Bayesian analysis of the three earliest RCTs (n = 141) found that patients on DOACs experienced fewer strokes or thromboembolic events (1/71 vs. 7/70; log OR -2.02 (95% CI, -4.53 to -0.31)) and fewer bleeding events (2/71 vs. 9/70; log OR -1.62 (95% CI, -3.43 to -0.26)) [6]. The 2024 JACC State-of-the-Art Review cautioned that these same three trials (n = 139 in their frequentist analysis) were insufficiently powered [4].

Since the AHA Scientific Statement, additional data have emerged. A 2024 meta-analysis of eight studies comprising 605 patients with LV thrombus after acute myocardial infarction found that DOACs were associated with an almost twofold higher likelihood of thrombus resolution compared with VKAs (pooled OR 1.95 (95% CI, 1.25-3.04); P = 0.003), a 70% lower risk of systemic embolism (pooled OR 0.30 (95% CI, 0.12-0.75); P = 0.01), and a 54% lower risk of bleeding (pooled OR 0.46 (95% CI, 0.26-0.84); P = 0.01) [7]. A 2025 updated systematic review and meta-analysis restricted to seven RCTs comprising 554 patients found no significant difference in complete LV thrombus resolution at three months (86% vs. 81%; risk ratio (RR) 1.01 (95% CI, 0.93-1.10); P = 0.76), nor in major or clinically significant bleeding (2.8% vs. 4.4%; RR 0.75 (95% CI, 0.26-2.19)) or the composite of stroke or thromboembolic complications (2.2% vs. 3.4%; RR 0.75 (95% CI, 0.12-4.25)) [8]. The largest of these trials, RIVAWAR, randomized 261 patients with post-myocardial infarction LV thrombus 2:1 to rivaroxaban 20 mg daily or warfarin and demonstrated comparable resolution at 12 weeks (95.8% vs. 96.6%), with a higher rate of complete resolution at four weeks in the rivaroxaban arm (20% vs. 8.3%; P = 0.017) [9]. The 2025 ACC/AHA/ACEP/NAEMSP/SCAI Guideline for the Management of Patients With Acute Coronary Syndromes acknowledges that observational studies and small RCTs suggest DOACs may be noninferior to VKAs with respect to mortality, stroke, or LV thrombus resolution, with an improved bleeding profile [10].

Critically for the present case, this entire randomized evidence base was generated in patients with preserved or only mildly impaired renal function. Every one of the seven RCTs included in the 2025 meta-analysis explicitly excluded patients with significant renal dysfunction, with exclusion thresholds of creatinine clearance below 50 mL/min in two trials, eGFR below 30 mL/min/1.73 m² in two trials, creatinine clearance below 15 mL/min in one trial, and unquantified "severe" or "advanced" renal disease in the remaining two [8]. Our patient, with an admission eGFR of 29 mL/min/1.73 m², would have been ineligible for enrollment in the majority of these trials. Consequently, no randomized data whatsoever inform anticoagulant selection for LV thrombus in the population he represents.

The clinical challenge in our patient is compounded by the renal dependence of DOAC elimination. DOACs are renally cleared to varying degrees-dabigatran approximately 80%, edoxaban approximately 50%, rivaroxaban approximately 33%, and apixaban approximately 27%-and dose adjustments based on renal function are critical to avoid either subtherapeutic anticoagulation or excessive bleeding risk [4]. The 2023 ACC/AHA/ACCP/HRS Atrial Fibrillation Guideline provides the most detailed renal-stratified DOAC recommendations available, though exclusively for the atrial fibrillation indication: DOACs are recommended preferentially over warfarin for CKD stage 3 (Class 1, Level of Evidence B-R); warfarin or labeled DOAC doses are considered reasonable for CKD stage 4 (Class 2a, Level of Evidence B-NR); and warfarin or evidence-based doses of apixaban may be considered for end-stage CKD or dialysis (Class 2b, Level of Evidence B-NR) [2]. In a prespecified renal-function analysis of the ARISTOTLE trial, apixaban maintained its efficacy and safety profile relative to warfarin across all levels of renal function [11]. However, these recommendations are specific to atrial fibrillation and cannot be directly extrapolated to LV thrombus, which involves a different pathophysiological mechanism of thrombus formation-stasis in a dysfunctional ventricle versus atrial stasis.

From the nephrology perspective, the Kidney Disease Outcomes Quality Initiative (KDOQI) US Commentary on the KDIGO 2024 Clinical Practice Guideline concurs with the recommendation of non-VKA oral anticoagulants in preference to VKAs for thromboprophylaxis in persons with CKD G1 to G4 and atrial fibrillation, while acknowledging greater uncertainty regarding safety and efficacy in more advanced stages of CKD and noting that dose adjustment for glomerular filtration rate is required, with particular caution at CKD G4-G5 [12]. Importantly, none of these renal-specific recommendations have been validated in the LV thrombus population. The situation is further complicated in AKI, where the Cockcroft-Gault equation used for DOAC dose adjustment presupposes a steady-state creatinine and is unreliable when renal function is changing. Our patient illustrates this concretely: a DOAC dose calculated on hospital day 1 at a creatinine of 2.6 mg/dL would have differed materially from one calculated on day 6 at 1.7 mg/dL, and no validated framework exists to determine which value should have governed dosing, or how frequently redosing should occur during recovery.

The clinical scenario described in this case is far from rare. In the Swedish Heart Failure Registry, CKD defined as an eGFR below 60 mL/min/1.73 m² was present in 45% of patients with HFrEF, 48% of those with mid-range ejection fraction, and 56% of those with preserved ejection fraction [13]. In a national Veterans Affairs cohort of 365,000 patients with heart failure and available ejection fraction data, KDIGO-defined CKD was associated with significantly higher adjusted risks of death and heart failure hospitalization across all ejection fraction categories [14]. Among 365,494 heart failure hospitalizations in the Get With The Guidelines-Heart Failure registry, 64% had a discharge eGFR below 60 mL/min/1.73 m² and 5% were receiving dialysis [15]. AKI is likewise common: a systematic review and meta-analysis of 37 studies found a pooled AKI incidence of 33% among patients with heart failure, substantially higher than the 3.2-9.6% reported among hospitalized general medical patients, and AKI more than tripled the risk of in-hospital mortality [16].

Estimates of LV thrombus prevalence in nonischemic cardiomyopathy vary widely by era and imaging modality. Historical two-dimensional echocardiographic series in dilated cardiomyopathy reported prevalence rates of roughly 10-30%, whereas contemporary cohorts report considerably lower figures, including 2.3% among more than 3,000 patients with dilated cardiomyopathy at a single tertiary center [17]. Nonetheless, the embolic consequences remain substantial. A nationwide analysis of the Japanese Registry of All Cardiac and Vascular Diseases-Diagnosis Procedure Combination (JROAD-DPC) database identified 1,037 analyzable patients with LV thrombus, of whom 826 (79.7%) had ischemic and 211 (20.3%) had nonischemic cardiomyopathy; while overall in-hospital rates of embolic stroke or systemic embolism did not differ significantly between groups, dilated cardiomyopathy carried a significantly higher adjusted OR for stroke or systemic embolism compared with ischemic cardiomyopathy (OR 2.6; 95% CI, 1.2-6.0; P = 0.022) [18]. A cardiac magnetic resonance (CMR)-based study found that independent predictors of LV thrombus in nonischemic cardiomyopathy included lower LVEF (hazard ratio (HR) 1.36 per 5% decrease), late gadolinium enhancement (LGE) presence (HR 6.30), and greater LGE extent (HR 1.33 per 5% increase), with a 12-month incidence of embolism of 8.7%, comparable to the 6.8% observed in ischemic cardiomyopathy [19]. Retrospective analyses cited in the AHA Scientific Statement have noted that thrombus recurrence or enlargement was associated with prothrombotic conditions including chronic renal failure, suggesting that patients with renal impairment may represent a higher-risk subgroup requiring particularly reliable anticoagulation [1].

Two major trials are actively recruiting to address the DOAC versus warfarin question for LV thrombus in the general population: the ARGONAUT trial (Anticoagulation Therapy in Non-device-related Intracardiac Thrombus; NCT05825573, planned n = 340), which randomizes patients to a VKA or one of apixaban, dabigatran, or rivaroxaban [4], and the RELEVENT trial (REsolution of LEft VENTricular thrombus; ACTRN12618001254280, planned n = 216), which will test noninferiority of any DOAC approved for stroke prevention in atrial fibrillation against warfarin [9]. Neither trial specifically addresses patients with renal impairment. Even when these trials report results, the renal impairment subgroup will likely remain unanswered, perpetuating the guideline gap illustrated by this case.

Perhaps the most practically significant aspect of this case is the five additional hospital days required solely for heparin-to-warfarin bridging. Had DOAC use been supported by evidence in this clinical context, the patient could have been discharged on an oral anticoagulant without parenteral bridging, potentially shortening the admission by more than half. Heart failure hospitalizations are the dominant driver of heart failure-related medical costs in the United States; a systematic review of US cost studies estimated median annual total medical costs of $24,383 per patient with heart failure, of which heart failure-specific hospitalization accounted for a median of $15,879 per patient, with per-hospitalization estimates varying widely across studies and rising with length of stay [20]. When extrapolated across the large population of patients with HFrEF and concurrent renal dysfunction, the aggregate healthcare expenditure attributable to mandatory warfarin bridging in this population is considerable. Extended hospitalization also exposes patients to nosocomial risks including hospital-acquired infections, venous thromboembolism from immobility, and medication errors. As demonstrated by our patient's INR trend (Table 3), achieving stable therapeutic anticoagulation with warfarin can be protracted, particularly in the setting of heart failure where hepatic congestion, fluctuating volume status, dietary changes, and drug interactions can all affect warfarin metabolism.

Until prospective data are available, clinicians encountering this common clinical intersection may consider the following pragmatic framework, which represents the authors' opinion rather than an evidence-based recommendation. In patients with LV thrombus and stable CKD stage 3, a DOAC may be considered with close monitoring, extrapolating from atrial fibrillation data while acknowledging the absence of LV thrombus-specific evidence. In patients with CKD stages 4-5, AKI, or renal function that has not reached steady state, warfarin with heparin bridging remains the more prudent choice given its hepatic metabolism, reliable INR monitoring, and reversibility. This framework, while not evidence-based for the LV thrombus indication specifically, may help guide shared decision-making until dedicated studies address this gap.

This case has several limitations inherent to a single-case report, including the inability to draw generalizable conclusions and the absence of long-term follow-up data regarding thrombus resolution, embolic events, or bleeding complications. With respect to the renal evaluation, no baseline creatinine was available, and although the trajectory of recovery supports a KDIGO-defined AKI, the elevated urine protein-to-creatinine ratio and the mild parenchymal heterogeneity on ultrasound mean that an underlying CKD unmasked by the acute illness cannot be excluded; repeat quantification of proteinuria after euvolemia was deferred to the outpatient setting. Regarding the cardiomyopathy, the etiologic evaluation was limited to clinical history, coronary angiography, and serum protein electrophoresis; thyroid function testing, iron studies, HIV serology, and antinuclear antibody testing were not obtained, serum free light chains were not performed, and endomyocardial biopsy was not undertaken, so a definitive etiologic diagnosis was not established. Cardiac magnetic resonance imaging was not performed, which could have provided more detailed thrombus characterization, myocardial tissue characterization, and prognostic information on LGE. The attribution of five excess hospital days to bridging alone reflects clinical judgment rather than a protocolized discharge-readiness assessment. The accompanying review was narrative rather than systematic, used a single primary database, and did not include a formal risk-of-bias assessment, so selection bias in the cited literature cannot be excluded.

## Conclusions

This case report and literature review highlight the absence of guideline-directed evidence for DOAC use in the treatment of LV thrombus in patients with AKI or significant renal impairment. The requirement for heparin-to-warfarin bridging prolonged our patient's hospitalization by five days, illustrating the tangible clinical and resource burden imposed by this guideline gap. While DOACs appear noninferior to warfarin for LV thrombus in the general population, these data have been generated almost exclusively in patients with preserved or mildly impaired renal function, and every randomized trial to date has explicitly excluded patients with moderate-to-severe renal dysfunction. No prospective study has evaluated DOAC safety and efficacy for LV thrombus in patients with CKD stage 4-5 or AKI, and renal-stratified DOAC dosing recommendations from atrial fibrillation guidelines have not been validated for this indication. Given the high prevalence of CKD and AKI among patients hospitalized with heart failure-the very population at greatest risk for LV thrombus-this evidence gap affects a substantial number of patients. Prospective studies incorporating dedicated renal impairment subgroups, including within ongoing trials such as ARGONAUT and RELEVENT, are needed. Such evidence could safely reduce hospital length of stay, decrease costs, and improve patient outcomes without compromising anticoagulation efficacy. Until such data are available, anticoagulant selection in this population should be individualized on the basis of clinical judgment and shared decision-making.
